# Heterochromatin: did H3K9 methylation evolve to tame transposons?

**DOI:** 10.1186/s13059-021-02550-5

**Published:** 2021-12-03

**Authors:** Manisha Kabi, Guillaume J. Filion

**Affiliations:** Department of Biological Sciences, University of Toronto Scarborough, 1265 Military Trail, Scarborough, M1C1A4 ON Canada

Chromatin is traditionally studied in the fields of DNA repair and gene regulation, not immunology. Yet, chromatin protects the genome against several types of viruses [1, 2], so it is part of the immune system of eukaryotes in a strict sense. Chromatin allows the host genome to shut down the transcription of some genes, irrespective of their sequence. In fact, it is so well designed for the task of controlling foreign genes that one may wonder whether the function of chromatin in immunity was its *raison d’être* [3].

The appearance of eukaryotes coincided with the expansion of a class of retrotransposons known as non-LTR [4]. Those transposable elements feature an aggressive copy-paste replication cycle that involves an RNA intermediate [3]. They are nearly absent from prokaryotic genomes, while they can represent a large fraction of eukaryotic genomes. In humans, for instance, the most abundant non-LTR retrotransposon is LINE-1 (long interspersed nuclear element 1), with approximately 85,000 full-length copies summing up to ~ 20% of the genome.

The fate of non-LTR retrotransposons is somehow bound with that of eukaryotes. It is thus plausible that some features of the early eukaryotic genomes conferred increased resistance to copy-paste mobile elements, protecting the organism against their harmful effects, but also creating an evolutionary niche for their sustained existence.

The chromatin of eukaryotes contains a specialized subtype called heterochromatin, loosely defined as genomic regions that are compacted and rich in repeated sequences, and where transcription is restrained [5]. Is heterochromatin responsible for the tolerance of eukaryotic genomes to retrotransposons?

In modern eukaryotes, the silencing functions of chromatin are carried out by three systems: the methylation of H3K9 (the lysine residue at position 9 of histone H3), the methylation of H3K27 (the lysine residue at position 27 of histone H3), and the methylation of cytosine in the genome [5]. Here, we will look in greater depth at H3K9 methylation because H3K27 methylation is mostly involved in the regulation of developmental genes (i.e., it is not part of constitutive heterochromatin) and cytosine methylation often changed function in the evolutionary history of eukaryotes.

**Fig. 1:**
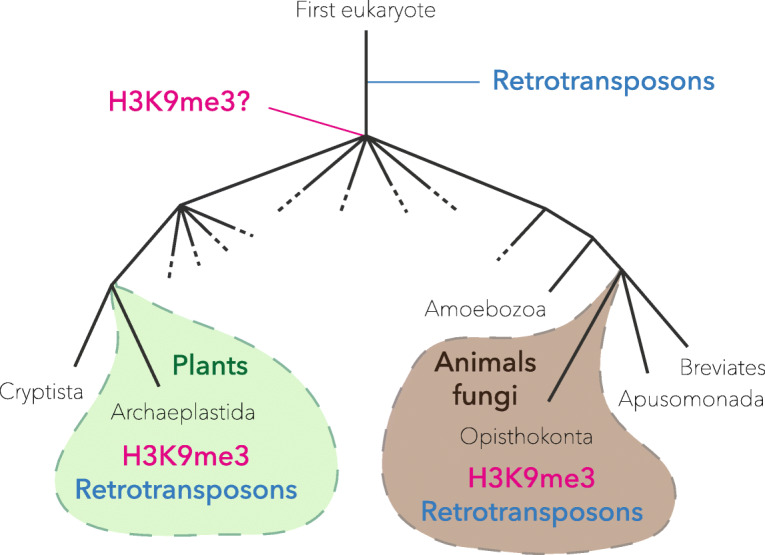
Partial phylogeny of eukaryotes. The genomes of plants, animals, and fungi contain the epigenetic mark H3K9me3 (the lysine residue at position 9 of histone H3 can be tri-methylated). H3K9me3 is thus present in the clades Archaeplastida and Opisthokonta, which have a common ancestor close to the base of all eukaryotes. This suggests that H3K9me3 was already present in early eukaryotes, around the time when retrotransposons appeared in their genomes (phylogeny adapted from [6], showing only the clades that are relevant for the argument)

Methylation of H3K9 is widespread among eukaryotes; more importantly, it is present in plants and animals, which belong to two distant clades called Archaeplastida and Opisthokonta, with a common ancestor near the base of all eukaryotes [6]. Either H3K9 methylation was present before the two lineages split, or it appeared multiple times independently. The H3K9 methyltransferase subfamily SUV39 is conserved in plants and animals [7], so it is likely that H3K9 methylation appeared only once, in the same evolutionary time frame as non-LTR retrotransposons (Fig. 1).

Some extant eukaryotic species do not have H3K9 methylation; for instance, the yeast *Saccharomyces cerevisiae* lost it in the course of its evolution. But when it is present, H3K9 methylation is found on repeated sequences and, in particular, on transposable elements [1]. Heterochromatin is the main barrier to the expression of transposable elements and endogenous retroviruses. In some modern eukaryotes, H3K9 methylation is also involved in differentiation and gene regulation [8], but the bulk of the mark is on repeated sequences in constitutive heterochromatin. Parsimony suggests that H3K9 methylation was already associated with repeated sequences in the genomes of early eukaryotes. Non-LTR retrotransposons must have been major contributors to the pool of repeats, so it is plausible that they were marked by H3K9 methylation and turned into heterochromatin.

However, H3K9 methylation is not intrinsically repressive. It acts as a signal that can be perpetuated through epigenetic feedback loops, but other proteins must be present in order to set up a domain where transcription is disfavored. In animals, the main repressor associated with H3K9 methylation is HP1 (heterochromatin protein 1), an adapter protein that interacts with the mark and recruits several repressors of transcription [5]. In plants, the function of HP1 is carried out by the non-homolog plant-only protein ADCP1 [9]. Plants have HP1-like proteins, but those are dedicated to binding methylated H3K27. A plausible scenario is that the H3K9 and H3K27 methylation systems were initially repressed by a single HP1-like repressor that had different fates in plants and animals, but the origins of those systems are mostly unclear.

H3K9-type heterochromatin is the main barrier to the expression of retroelements and endogenous retroviruses [1]. In addition, it is involved in kinetochore assembly and in the suppression of recombination on repeated sequences [1]. Interestingly, all the functions of H3K9 heterochromatin are associated with repeated sequences. Given their prevalence, those repeats were most likely non-LTR retrotransposons in early eukaryotes, so it is reasonable to assume that ancestral heterochromatin was already targeted to non-LTR retrotransposons. The function of ancestral heterochromatin remains mysterious, but the scenarios envisioned here suggest that it created a special chemical environment around non-LTR retrotransposons.

A similar argument may have been made with the cytosine methylation system, which is also involved in the silencing of retroelements. The machinery of cytosine methylation is conserved between plants and animals [10]. Interestingly, cytosine methylation is a common component of immunity in prokaryotes, at work in restriction-modification systems to detect non-self DNA. Perhaps the pressure to silence non-self genes was a common theme of early epigenetic mechanisms [3].

In conclusion, the rise of non-LTR retrotransposons at the origin of eukaryotes and the omnipresent association between H3K9 methylation and repeated sequences in modern eukaryotic genomes suggest that heterochromatin allowed eukaryotes to repress transposons. One can surmise that the capacities of the system were later expanded to meet the demands for complex gene regulation, but heterochromatin kept its role as the main barrier against the uncontrolled proliferation of transposable elements. If this view is correct, it implies that early eukaryotes acquired the capacity to identify transposable elements inserted in their genomes. Those putative ancient and conserved mechanisms have not yet been discovered.
